# Aerobic but not thermoregulatory gains following a 10‐day moderate‐intensity training protocol are fitness level dependent: A cross‐adaptation perspective

**DOI:** 10.14814/phy2.14355

**Published:** 2020-02-15

**Authors:** Alexandros Sotiridis, Tadej Debevec, Urša Ciuha, Adam C. McDonnell, Tinkara Mlinar, Joshua T. Royal, Igor B. Mekjavic

**Affiliations:** ^1^ Department of Automation, Biocybernetics and Robotics Jozef Stefan Institute Ljubljana Slovenia; ^2^ Faculty of Sports University of Ljubljana Ljubljana Slovenia; ^3^ Jozef Stefan International Postgraduate School Ljubljana Slovenia

**Keywords:** aerobic performance, fitness level, thermoregulation, training effect

## Abstract

Moderate‐intensity exercise sessions are incorporated into heat‐acclimation and hypoxic‐training protocols to improve performance in hot and hypoxic environments, respectively. Consequently, a training effect might contribute to aerobic performance gains, at least in less fit participants. To explore the interaction between fitness level and a training stimulus commonly applied during acclimation protocols, we recruited 10 young males of a higher (more fit‐MF, peak aerobic power [VO_2peak_]: 57.9 [6.2] ml·kg^−1^·min^−1^) and 10 of a lower (less fit‐LF, VO_2peak_: 41.7 [5.0] ml·kg^−1^·min^−1^) fitness level. They underwent 10 daily exercise sessions (60 min@50% peak power output [W_peak_]) in thermoneutral conditions. The participants performed exercise testing on a cycle ergometer before and after the training period in normoxic (NOR), hypoxic (13.5% F_i_O_2_; HYP), and hot (35°C, 50% RH; HE) conditions in a randomized and counterbalanced order. Each test consisted of two stages; a steady‐state exercise (30 min@40% NOR W_peak_ to evaluate thermoregulatory function) followed by incremental exercise to exhaustion. VO_2peak_ increased by 9.2 (8.5)% (*p* = .024) and 10.2 (15.4)% (*p* = .037) only in the LF group in NOR and HE, respectively. W_peak_ increases were correlated with baseline values in NOR (*r* = −.58, *p* = .010) and HYP (*r* = −.52, *p* = .018). MF individuals improved gross mechanical efficiency in HYP. Peak sweat rate increased in both groups in HE, whereas MF participants activated the forehead sweating response at lower rectal temperatures post‐training. In conclusion, an increase in VO_2peak_ but not mechanical efficiency seems probable in LF males after a 10‐day moderate‐exercise training protocol.

## Graphical abstract

Whether a substantial training effect can be potentiated by short‐term acclimation protocols that include exercise sessions remains unresolved. Here, we demonstrate that a training effect might contribute to aerobic gains only in individuals of a lower fitness level that participate in heat‐acclimation protocols.

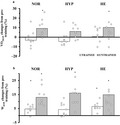

## 1 INTRODUCTION

Acute exercise creates a competition between the exercising muscles and skin for the prevailing cardiac output; the former requiring its share of the cardiac output for oxygen delivery and the latter for delivery of heat—to be eventually dissipated to the environment—from the core via the blood circulation. Exercise training reduces this competition by not only enhancing convective oxygen delivery (Saltin, Blomqvist, Mitchell, Johnson Jr, Wildenthal, & Chapman, 1968), but also rendering the sweating response more efficient (Nadel, Pandolf, Roberts, & Stolwijk, 1974). Training in the heat augments the exercise‐induced improvements in temperature regulation (Lorenzo & Minson, 2010; Nadel et al., 1974; Roberts, Wenger, Stolwijk, & Nadel, 1977) and subsequently enhances aerobic performance in hot environments (Racinais, Periard, Karlsen, & Nybo, 2015). In contrast, adaptation to hypoxia does not require the inclusion of exercise (Siebenmann, Cathomen, et al., 2015a). Nevertheless, heat (HeA)‐ and hypoxic‐acclimation (HyA) protocols might include exercise during exposure to the respective environments (Bonne, Lundby, et al., 2014b; Lorenzo, Halliwill, Sawka, & Minson, 2010). In the former case, exercise is utilized as a means to facilitate rapid increases in core temperature (T_c_) which serve as the key stimulus for heat adaptation (Regan, Macfarlane, & Taylor, 1996). The incorporation of exercise sessions into acclimation protocols renders it difficult to distinguish the effect of physiological adaptation to the environmental stressor under investigation from that of the exercise training per se. Control groups are not always included in acclimation studies, thus the separation of the training effect from the acclimation effect is poorly characterized. This is now of paramount importance in studies investigating the manner in which the adaptation to one stressor (i.e., heat) may provide performance benefits when exposed to another stimulus (i.e., hypoxia). This concept of cross‐adaptation was initially proposed by Hessemer, Zeh and Bruck (1986), and more recently by Maloyan et al. (2005); the former basing their suggestion on physiological responses at the systems level to cold and heat exposure, and the latter providing evidence of cross‐adaptation at the cellular level. Many groups have attempted to provide support for the theory of cross‐adaptation at the systems level (Ely, Lovering, Horowitz, & Minson, 2014; Lee, Miller, James, & Thake, 2016; White et al., 2016). We, too, have addressed this issue, and contrary to most of the reported studies, found sparse evidence of cross‐adaptation (Sotiridis, Debevec, Ciuha, Eiken, & Mekjavic, 2019a; Sotiridis et al., 2018; Sotiridis, Miliotis, Ciuha, Koskolou, & Mekjavic, 2019b). Several factors may have contributed to this. One factor which has largely been ignored is that of exercise. Acclimation studies mostly include exercise training to augment the process of HeA and/or HyA, ignoring the fact that the exercise is also a stressor. Exercise training, although of moderate intensity when used to promote acclimation, imposes a degree of adaptation that may be reflected in the system response to exercise in hot and hypoxic environments. The elucidation of this cross‐over effect of physical training on performance in the heat and simulated altitude was the main aim of this study. This study was, therefore, not designed to compare the effect of training on high‐fit and low‐fit individuals. The novelty of the study rather lies in its examination of any evidence of cross‐adaptation to the stressors of heat and hypoxia, and additionally whether this adaptive response is modified by fitness level.

Short‐term (e.g., 10‐day) training protocols employed in untrained individuals are believed to potentiate heat dissipation responses owing to the training‐induced repeated elevations of T_c_ (Avellini, Shapiro, Fortney, Wenger, & Pandolf, 1982; Hessemer et al., 1986). Augmented heat dissipation might be of central or peripheral origin and, respectively, expressed as a lower sweating threshold (Nadel et al., 1974) or a higher gain of the sweating response (Roberts et al., 1977). Consequently, decrements in exercise heart rate (HR), rectal temperature (T_re_), and skin temperature are observed, whereas maximal skin wettedness can also be enhanced after longer protocols (Ravanelli, Coombs, Imbeault, & Jay, 2018). Surprisingly, no study to date has examined whether these phenotypic adaptations in an untrained population are translated into a lower decrement in aerobic performance in the heat, compared with thermoneutral conditions. Furthermore, the training level seems to be correlated with the decrement in aerobic performance in hypoxia (Koistinen, Takala, Martikkala, & Leppaluoto, 1995; Martin & O'Kroy, 1993) underlined by the exaggerated reduction in capillary oxyhemoglobin saturation (S_p_O_2_) in the trained individuals (Mollard et al., 2007).

Common to acclimation studies is that the efficiency of acclimation protocols is evaluated on the basis of physical performance. Specifically, aerobic exercise performance is determined by peak aerobic power (VO_2peak_), gross mechanical efficiency (GME), and the lactate/anaerobic threshold (Bassett Jr & Howley, 2000). In recent HeA studies (Karlsen et al., 2015; Keiser et al., 2015; Lorenzo et al., 2010), the control groups undergoing training at normoxic thermoneutral conditions did not improve any of the aerobic performance measures. Interestingly, studies including only an experimental group have reported a higher GME after HeA incorporating exercise training (Sawka, Pandolf, Avellini, & Shapiro, 1983; Sotiridis, Debevec, et al., 2019a), but not after a training protocol of a similar duration held in a temperate environment (Sawka et al., 1983). As such, any positive results observed in the experimental groups should have resulted from the interactive effect between training and the heat. Otherwise, the inefficiency of the acclimation protocol could be attributed to the high fitness level of the participants (Karlsen et al., 2015). We did not seek to provide the participants with a training stimulus that would stimulate cardiovascular adaptations. On the contrary, we decided to reproduce an exercise intensity of 50% peak power output (W_peak_) that is usually selected for fixed‐workload HeA protocols (Lorenzo et al., 2010). Secondly, this exercise intensity has been proposed (Gibson, Willmott, James, Hayes, & Maxwell, 2017) and selected (Sotiridis, Debevec, et al., 2019a) to potentiate the targeted increase in T_c_ during the initial 30‐min stage of controlled‐hyperthermia HeA protocols. Finally, given the fact that recent controlled‐hyperthermia protocols (Gibson et al., 2015; Rendell et al., 2017; Sotiridis, Debevec, et al., 2019a) have reported an average power output of ~ 30% W_peak_, a training intensity that would provide the participants with a similar training volume/mechanical strain, but over a lower duration (60 min instead of 90 min) is preferable.

Based on the above, there is a plethora of studies investigating the separate effects of HeA and HyA whereby the process of acclimation is augmented by the inclusion of physical exercise. To date, whether training per se impacts on exercise performance in hot and hypoxic environments remains unclear. An additional novelty of this study is the assessment of the interactive effects of a 10‐day moderate‐intensity training protocol and baseline fitness level on aerobic performance and thermoregulatory responses in normoxic, hypoxic, and hot conditions. We hypothesized that aerobic performance as well as thermoregulatory responses would be facilitated by the rather low‐intensity training protocol only in the untrained individuals across environmental conditions.

## 2 METHODS

### 2.1 Participants

Twenty healthy young male volunteers (age = 23.5 [2.6] years) were recruited to participate in the study. Inclusion criteria included healthy male participants between 18 and 30 years of age, nonsmokers, and medication free. All participants were near sea–level residents and had not been exposed to altitudes > 1,500 m or temperatures > 30°C for at least 1 month prior to the start of the study which was held in November and December. None had a history of any cardiorespiratory or hematological diseases. Participants were instructed to abstain from caffeine and alcohol consumption during the course of the entire study. They were also instructed to record their diet the night before the first test and they were requested to consume the same meals during the nights preceding the remaining exercise tests. Participants confirmed that they had consumed a light breakfast before attending the daily training sessions. They were extensively informed regarding the study protocol as well as the potential risks involved. During the prescreening procedure the participants completed a graded exercise test on a cycle ergometer to establish their normoxic VO_2peak_ and W_peak_. Aerobic fitness was defined based on VO_2peak_ values. A VO_2peak_ lower than 45 ml kg^−1^ min^−1^ or higher than 55 ml kg^−1^ min^−1^ was deemed as a prerequisite for the participation in the lower (less fit‐LF) or higher (more fit‐MF) fitness group, respectively, in line with the values reported in previous studies (Jay, Bain, Deren, Sacheli, & Cramer, 2011; Montero & Lundby, 2017). To further ensure that VO_2peak_ reflected the actual participants’ activity level, LF participants were also required not to participate in any organized sports. Cycling and walking for commuting purposes were allowed. Accordingly, MF participants were performing endurance‐type activities (running, cycling, and swimming) multiple times per week. Participants were informed that the aim of the study was to investigate the effect of a 10‐day training protocol on aerobic performance in young males. However, they were blinded to the fact that the data would be analyzed in groups according to their fitness level. Participant baseline characteristics are presented in Table 1. For the participants who were included in the study, the measured normoxic W_peak_ was used to determine 1) the individual steady‐state workloads during the pre‐ and post‐HA tests (40% of the W_peak_ attained during the preliminary test) and 2) the training intensity to be used during the 10‐day training protocol. The protocol was approved by the National Committee for Medical Ethics at the Ministry of Health (Republic of Slovenia, no. 0120‐494/2018/9) and conformed to the Declaration of Helsinki guidelines. Prior to the onset of the experiments, informed oral and written consents were obtained from all participants.

**Table 1:** Participants’ baseline characteristics

Group	More fit (*n* = 10)	Less fit (*n* = 10)
Age (years)	23 (2)	25 (3)
Height (cm)	180 (5)	179 (3)
Mass (kg)	74 (3)	85 (14)*
BSA (m^2^)	1.96 (0.08)	2.05 (0.17)
Body fat (%)	9.2 (2.3)	16.3 (4.9)*
VO_2peak_ (ml·kg^−1^·min^−1^)	58 (6)	42 (5)*
W_peak_ (W)	364 (35)	309 (46)*
W_peak_ (W/kg)	4.9 (0.5)	3.6 (0.4)*

Values are means (*SD*).

Abbreviations: BSA, body surface area; VO_2peak_, peak aerobic power, W_peak_ peak power output.

^∗^Significant difference compared with the More Fit group

### 2.2 Study design

The study consisted of three parts: pretraining exercise testing, a 10‐day exercise training program, followed by the post‐training exercise testing. During exercise testing, the participants completed the same maximal exercise performance test in three different environmental conditions (thermoneutral normoxic, thermoneutral hypoxic, and hot normoxic) on separate but consecutive days. The order of the exercise tests was randomized and counterbalanced between participants, whereas the order of the tests within participants was the same pre‐ and post‐training. All tests were performed the same time of the day for a given participant (±1 hr). Exercise training sessions were held during morning hours (9:00–12:00). Participants were given a 24‐hr rest prior to and following the 10‐day training period.

### 2.3 Exercise training

All participants underwent 10 × 60 min supervised cycling sessions over 10 days. Training intensity was similar in relative terms for all individuals, set at 50% of the W_peak_ calculated from the individual W_peak_ obtained during the preliminary graded normoxic exercise test. Participants were only informed of the time remaining to the end of the session and were allowed to drink water ad libitum during each training session. Exercise HR and S_p_O_2_ were assessed using a finger‐pulse oximeter (Wristox 3,100 Nonin, Plymouth) at 5‐min intervals. Ratings of perceived exertion (RPE; 6–20 [Borg, 1970]) were also recorded at 5‐min intervals. Ambient temperature (T_a_) was maintained at 24℃. The exercise room was well ventilated, ensuring normoxic and normocapnic conditions during training. The participants performed all training sessions at the same time of the day. No other exercise training was allowed during the study. The sessions were supervised by at least two researchers in order to record the training data and to ensure that all participants maintained the desired workloads at all times.

### 2.4 Exercise testing

The protocol and the environmental conditions of the exercise trials have been extensively described previously (Sotiridis, Debevec, et al., 2019a; Sotiridis et al., 2018; Sotiridis, Miliotis, et al., 2019b). Briefly, all the pre‐ and post‐training trials were conducted in a laboratory situated at 300 m above sea level (Ljubljana). The trials were conducted on a cycle ergometer (Daum, Electronic) and comprised two stages; a 30‐min steady‐state exercise immediately followed by an incremental exercise to exhaustion. During exercise, each participant pedaled at a preferred cadence (between 60 and 90 rpm), which they maintained via visual and verbal feedback throughout the trial. Before (Pre) and after (Post) the 10‐day training protocol, participants conducted three trials on three consecutive days. In the normal temperature and normoxic condition (NOR), participants breathed room air (pre: P_i_O_2_ = 143.7 [0.8] mmHg, post: P_i_O_2_ = 143.4 [0.7] mmHg) and exercised in thermoneutral conditions (pre: T_a_ = 23.2 [0.7] ℃ and relative humidity [RH] = 47.2 [2.2]%, post: T_a_ = 23.2 [0.5] ℃ and RH = 46.6 [5.9]%). In the hypoxic condition (HYP) simulating an altitude of 4,000 m, they inspired a hypoxic gas mixture (pre: P_i_O_2_ = 92.2 [1.5] mmHg, post: P_i_O_2_ = 93.2 [1.2] mmHg) and exercised in thermoneutral conditions (pre: T_a_ = 22.8 [0.5] ℃ and RH = 51.2 [1.2]%, post: T_a_ = 22.5 [0.6] ℃ and RH = 51.5 [1.3]%). In the hot condition (HE), the participants inspired room air (pre: P_i_O_2_ = 142.6 [1.7] mmHg, post: P_i_O_2_ = 142.7 [1.8] mmHg), but exercised in a hot environment (pre: T_a_ = 34.1 [0.9] ℃, RH = 48.1 [4.2] %, post: T_a_ = 34.1 [1.1] ℃ and RH = 49.8 [3.0]%). The wind speed was <0.5 m/s. As stated earlier, the order of the exercise trials was randomized and counterbalanced. A resting day was scheduled immediately before and after the training protocol to minimize the contribution of fatigue during the exercise tests. Body composition was assessed on days 1 and 10 of the training protocol by taking measurements of seven skinfolds (chest, thigh, triceps, subscapular, suprailiac, abdominal, and axillary) and using the equation of Jackson and Pollock (1978) for the calculation of body fat percentage.

A metabolic cart (Quark CPET, Cosmed) was used to obtain the breath‐by‐breath respiratory responses during the exercise trials. Metabolic energy expenditure was calculated during submaximal exercise using the following equation (Nishi, 1981):

$$M\;\left(W\right)={\dot{V}O}_{2}\frac{\left(\frac{\text{RER}-0.7}{0.3}\right)e_{c}+\left(\frac{1-\text{RER}}{0.3}\right)e_{f}}{60},$$

where RER respiratory exchange ratio, VO_2_ oxygen uptake (ml**/**min), and e_c_ and e_f_ are the caloric equivalents per liter of oxygen for the oxidation of carbohydrates (21.13 kJ) and fats (19.62 kJ), respectively. Metabolic heat production (H_prod_) was estimated by subtracting the rate of mechanical work from metabolic energy expenditure. The second ventilatory threshold (VT2) was defined as the oxygen uptake that corresponded to the deflection point of the end‐tidal CO_2_ confirmed by the nadir in the V_E_/VCO_2_ when plotted as functions of the workload during the incremental test (Binder et al., 2008). Before each trial, the O_2_ and CO_2_ analyzers were calibrated using two different gas mixtures, and the pneumotachograph was calibrated with a 3‐L syringe, in accordance with the manufacturer's recommendation.

HR was derived from electrocardiographic recordings with electrodes in a precordial position. Cardiac output (CO) was determined using electrical impedance cardiography (Physioflow Q‐Link, Manatec Biomedical). The method measures changes in thoracic impedance during cardiac ejection to estimate stroke volume (SV). Six electrodes were placed at the base of the neck and on the xiphoid process. Electrical impedance cardiography has been validated against the direct Fick method during maximal incremental exercise in healthy participants (Siebenmann, Rasmussen, et al., 2015b). S_p_O_2_ was recorded using an ear‐pulse oximeter (Wristox_2_ 3150 Nonin, Plymouth). Ratings of perceived exertion were obtained using a Borg scale (6–20). During all trials the participants wore only shorts and athletic shoes. The body mass was measured before and after each exercise testing on a weighing scale (±0.05 kg) (TPT 5N, Libela ELSI, Celje).

Rectal temperature (T_re_) was measured with a thermistor probe (MSR 145, Henggart) inserted 10 cm beyond the anal sphincter. Skin temperatures were measured at six sites (chest, arm, thigh, calf, forearm, and fingertip) using thermistors (MSR 147WD, Henggart) attached to the skin with a single piece of adhesive tape. The assessment of calf, thigh, chest, and arm skin temperature enabled the calculation of weighted mean skin temperature (T_sk_) (Ramanathan, 1964). The forearm–fingertip temperature difference (ΔΤ_f‐f_) has been validated as an index of skin vasomotor tone during steady‐state exercise (Keramidas, Geladas, Mekjavic, & Kounalakis, 2013). Minute mass flow of secreted sweat (ṁ_sw_) was measured with a ventilated capsule placed on the forehead. Forced evaporation of sweat under the capsule (surface area 4.8 cm^2^) was achieved by a constant air flow (1.0 L/min) through the capsule. Minute mass flow of secreted sweat was estimated each minute from the difference between the temperature and the humidity of inflowing and outflowing air. Air temperature was measured with thermistors (LM35, National Semiconductor Corp.) and the relative humidity with capacitance hygrometers (Valvo air humidity sensor, Valvo‐Philips GmbH). Fluid intake was restricted during each exercise trial.

#### 2.4.1 Steady‐State and incremental exercise

Following a 2‐min rest period, participants commenced pedaling on the cycle ergometer. During the first 2 min of the exercise, the load was set at 90 W (warm‐up). Thereafter, they cycled for 30 min at a steady‐state work rate equivalent to 40% of their NOR W_peak_ attained during the preliminary testing. The absolute and not the relative external workload was the same in all three exercise trials (the same power output was employed for a specific participant across environmental conditions). Immediately following the 30‐min steady‐state exercise protocol, participants performed an incremental‐load exercise to exhaustion. The initial work rate was set at 100 W (2 min), thereafter increasing by 20 W every minute until volitional exhaustion (inability to maintain the cycling cadence above 60 rpm). During each trial, S_p_O_2_ and sweat rate data were recorded at minute intervals, whereas HR, CO, and SV were measured every 10 s. RPE values were recorded at 5‐min and 2‐min intervals during the steady‐state and the incremental‐load exercise, respectively. Attainment of VO_2peak_, defined as the highest VO_2_ averaged over 30 s, was confirmed when participants met at least three of the following criteria, listed in order of priority: (a) severe fatigue or exhaustion leading to inability to maintain exercise at a given work rate (cycling cadence lower than 60 rpm), (b) a plateau in VO_2_, as indicated by the breath‐by‐breath values, despite an increase in power output (c) a subjective RPE near maximal (Borg scale rating greater than 17) and (d) respiratory quotient greater than 1.10. W_peak_ was calculated according to the following equation:

$$W_{\text{peak}}=\text{work rate of last stage completed}\;+\;\left[\left(\text{work rate increment}\right)\times \;\left(\text{time into current stage}/60\right)\right].$$

### 2.5 Hematology

Blood samples were obtained from the antecubital vein on the mornings of the day of the first exercise test, training days 2 and 6, and the resting day post‐training using standard venipuncture technique at the testing site. All samples were assayed in duplicate.

#### 2.5.1 Erythropoietin

Ten milliliter of whole blood were collected into standard serum tubes without activators (BD Vacutainer, BD‐Plymouth). Samples were subsequently put on ice for 1 hr to clot before being centrifuged at 1,600***g*** for 10 min so that serum was obtained. Serum samples were then aliquoted into 1 ml tubes and kept on −20℃ until further analysis. The erythropoietin was determined in 200 µl of serum using a two‐site enzyme‐linked immunosorbent assay (ELISA) (IVD, Erythropoietin ELISA, IBL). The quantification of the optical density was performed on a microplate reader (Biotek ELX 808, Bio‐tek Instruments, Winooski) set at 405 and 450 nm. The sensitivity of the measurement was 1.1 mIU/ml for EPO.

#### 2.5.2 Hct, Hb, and vascular volumes

Three ml of venous blood were additionally collected to determine Hb concentration and Hct using an automated hematology analyzer (ABX Micros ES 60, Horiba medical Kyoto, Japan). Pretraining resting vascular (blood, plasma, and erythrocyte) volumes were calculated from body surface area by the equations of Sawka, Young, Pandolf, Dennis, and Valeri (1992) and post‐training volumes were calculated by correcting that initial values for the percent change in plasma volume (Strauss, Davis, Rosenbaum, & Rossmeisl, 1951) as well as total blood and erythrocyte volume (Dill & Costill, 1974).

#### 2.5.3 Intracellular heat‐shock proteins (HSP70 and 90)

Twelve ml of whole blood was collected into Ficoll cell preparation tubes (CPTs) (BD Vacutainer CPT, BD, Becton Drive). Samples were followingly centrifuged at 1,500***g*** for 12 min. Four milliliter of plasma and 2 ml of phosphate buffer saline (PBS) with mononuclear cells (mostly monocytes and leukocytes) were obtained. After plasma was discarded, cells were washed with PBS twice. After each washing step, cells in PBS were centrifuged. One ml of extraction buffer together with a protease inhibitor cocktail tablet was added to the remaining cell pellet. After 30 min of incubation on ice, samples were centrifuged at 20.000 ***g*** for 10 min to break the cell pellets. The supernatant was then transferred into other sample tubes and the resultant cell lysates were frozen at −20℃ for later analysis. Intracellular Heat‐Shock Proteins (HSP) 70 and 90 concentration were determined by standard sandwich ELISA (Enzo Life Sciences Inc.) in 50 and 100 μl of cell lysates, respectively. Optical density was quantified on a microplate reader (Biotek ELX 808, Bio‐tek Instruments) set at 450 nm and corrected at 630 nm. The sensitivity of the measurement was 0.2 ng/ml and 0.05 ng/ml for HSP70 and HSP90, respectively.

### 2.6 Data analysis

Statistical analyses were performed using Statistica 5.0 (StatSoft). Hemodynamic variables (CO, HR, and SV) collected during steady‐state exercise were analyzed for the last 5 min of the steady‐state exercise, unless stated otherwise. The dependent thermoregulatory variables of H_prod_, sweating response (gain, threshold, and maximal capacity) as well as all temperature data were analyzed only for the 30 min of the submaximal exercise using a mixed three‐way ANOVA with the nonrepeated factor of group (two levels: MF and LF) and the repeated factors of environmental condition (three levels: NOR, HYP, and HE) and time (two levels: pre and post). Submaximal and peak values of cardiorespiratory and hemodynamic variables as well as several exercise performance variables (GME, VT2, and W_peak_) were analyzed using again a similar mixed three‐way ANOVA. Hematological variables were analyzed using a mixed two‐way ANOVA with the nonrepeated factor of group (two levels: MF and LF) and the repeated factor of time (four levels: pre, day 2, day 6, and post). When ANOVA revealed a significant F‐ratio for interaction and/or main effect, pairwise comparisons were performed with the Tukey honestly significant difference post hoc test. In the event that the statistical analysis revealed a non‐normal distribution of the data, a nonparametric test was performed. Such was the case for S_p_O_2_, ΔΤ_f‐f_, plasma erythropoietin concentration (EPO), and heat‐shock proteins (HSP70 and HSP90), which were analyzed using the Friedman nonparametric test. Planned comparisons were carried out using Kruskal–Wallis or Wilcoxon tests to locate specific differences. Baseline sweating has an approximate slope of zero, which changes in abrupt monotonic fashion in response to heat stress. We used segmented regression to identify the breakpoint in the forehead sweating response‐T_re_ curve (Cheuvront et al., 2009). The gain of the localized forehead sweating response was determined as the slope of the linear portion of the relationship between sweating response and T_re_ using least square linear regression. The reported values are averages of the individual thresholds and slopes. Cardiac output was standardized for body surface area by conversion to cardiac index. Linear regressions were also used to determine the Pearson's product‐moment correlation coefficients (*r*) between initial W_peak_ and VO_2peak_ and their respective percent post‐training changes or the percentage changes in maximal cardiac output (CO_max_). Correlation size was interpreted using the correlation classification of Hopkins, Marshall, Batterham, and Hanin (2009): trivial (*r* < .1), small (.1 < *r* < .3), moderate (.3 < *r* < .5), large (.5 < *r* < .7), very large (.7 < *r* < .9), nearly perfect (*r* > .9), and perfect (*r* = 1.0). Data are presented as mean (*SD*) unless indicated otherwise. The alpha level of significance was set a priori at 0.05.

## 3 RESULTS

### 3.1 Exercise training

MF individuals were exercising at a higher workload during the training sessions (*p* < .01). HR was lower from day 8 onwards (*p* < .05) compared with day 1 but did not differ between groups (*p* = .13). LF individuals tended to have a higher body mass across time (*p* = .064, Table 2). Participants across groups had a lower body mass post‐training (main effect of time: *p* = .038). Body fat percentage was lower in the MF group across time (*p* < .001) with both groups displaying a lower body fat percentage post‐training (MF: 9.2 [2.3] vs. 8.4 [2.0]%, *p* < .01, LF: 16.3 [4.9] vs. 14.8 [4.9]%, *p* < .001).

**Table 2:** Maximal cardiorespiratory responses and peak power output during the incremental exercise to exhaustion pre‐ and post‐training for the more fit (*n* = 10) and less fit (*n* = 10) groups in normoxic, hypoxic, and the heat tests

More Fit	Pretraining			Post‐training		
	NOR	HYP	HE	NOR	HYP	HE
VO_2peak_ (ml·kg^−1^·min^−1^)	58.3 (6.3)	47.8 (3.8)†	52.9 (4.9)	56.3 (5.5)‡†	45.7 (4.6)†	53.1 (5.8)
VO_2peak_ (L·min^−1^)	4.44 (0.53)‡†	3.62 (0.31)†	4.02 (0.32)	4.26 (0.43)‡	3.46 (0.34)†	4.04 (0.38)
W_peak_ (W)	359 (44)‡†	302 (31)†	331 (38)	373 (47)^*†‡^	314 (34)†	352 (38)*
W_peak_ (W/kg)	4.71 (0.52)‡†	3.98 (0.43)†	4.36 (0.50)	4.93 (0.58)^*†‡^	4.16 (0.54)^*†^	4.64 (0.56)*
CO_max_ (L·min^−1^)	23.6 (3.8)	23.2 (2.8)	24.5 (3.2)	25.2 (4.3)	23.4 (3.4)	25.0 (3.0)
Peak Cardiac index (L·min^−1^·m^−2^)	12.0 (1.7)	11.9 (1.3)	12.5 (1.5)	12.9 (2.0)	12.0 (1.6)	12.8 (1.5)
HR_peak_ (bpm)	192 (6)	186 (9)†	194 (5)	188 (7)‡	180 (9)†	190 (7)
SV_peak_ (ml)	123 (21)	125 (17)	127 (18)	134 (23)	131 (23)	131 (18)
Second ventilatory threshold (ml·kg^−1^·min^−1^)	46.5 (7.0)	38.9 (2.8)	42.5 (4.2)	45.0 (6.2)‡	37.6 (2.9)†	43.9 (3.4)
V_E peak_ (L·min^−1^)	186 (19)	194 (10)†	178 (15)	186 (15	182 (13	185 (16)
Body mass (kg)	76.2 (5.1)	75.9 (5.1)	76.2 (4.9)	75.8 (4.8)	75.9 (5.1)	76.1 (4.6)
Less Fit						
VO_2peak_ (ml·kg^−1^·min^−1^)	41.5 (6.0)§‡	35.8 (4.9)§†	40.0 (7.0)§	44.9 (4.1)^*§‡^	38.0 (5.2)†	43.2 (4.0)*
VO_2peak_ (ml·kg^−1^·min^−1^)	3.50 (0.53)§‡	3.02 (0.27)†	3.36 (0.48)	3.77 (0.42)^*‡^	3.17 ( 0.23)†	3.62 (0.41)*
W_peak_ (W)	293 (45)‡†	250 (33)†	277 (48)	328 (41)^*‡†^	286 (23)^*†^	311 (39)*
W_peak_ (W/kg)	3.47 (0.50)§‡†	2.97 (0.51)†	3.29 (0.59)	3.91 (0.56)^*‡†^	3.43 (0.51)^*†^	3.72 (0.50)*
CO_max_ (L·min^−1^)	23.5 (3.1)	21.1 (2.2)	23.5 (3.8)	25.6 (3.0)	24.3 (3.2)*	24.9 (3.8)
Peak Cardiac index (L·min^−1^·m^−2^)	11.5 (1.5)	10.3 (1.3)	11.5 (1.8)	12.6 (1.5)	11.9 (1.3)	12.0 (1.5)
HR_peak_ (bpm)	189 (11)	187 (12)†	193 (15)	187 (8)	182 (9)†	189 (8)
SV_peak_ (ml)	124 (15)	113 (12)	121 (16)	137 (18)	134 (17)*	133 (22)
Second ventilatory threshold (ml·kg^−1^·min^−1^)	32.6 (4.8)§‡†	30.9 (3.9)†	32.5 (6.2)§	33.7 (3.3)§‡	30.8 (3.3)†	34.2 (3.7)§
V_E peak_ (L·min^−1^)	141 (20)‡	144 (27)‡	137 (17)‡	160 (20*	164 (24)*	154 (16)*
Body mass (kg)	85.4 (14.3)	85.6 (14.2)	85.5 (13.9)	84.9 (13.3)	84.9 (13.6) *	84.7 (13.5) *

Note

VO_2peak_ maximal oxygen uptake, W_peak_ peak power output, CO_max_ maximal cardiac output. HR_peak_ heart rate corresponding to maximal cardiac output, SV_peak_ stroke volume corresponding to maximal cardiac output. Values are mean (*SD*)

* Significant difference to pretraining values

^†^ Significant difference to heat values

^‡^ Significant difference to hypoxic values

^§^ Significant difference to More Fit participants, *p* < .05

### 3.2 Aerobic capacity and cardiovascular responses during maximal exercise

**Figure 1:**
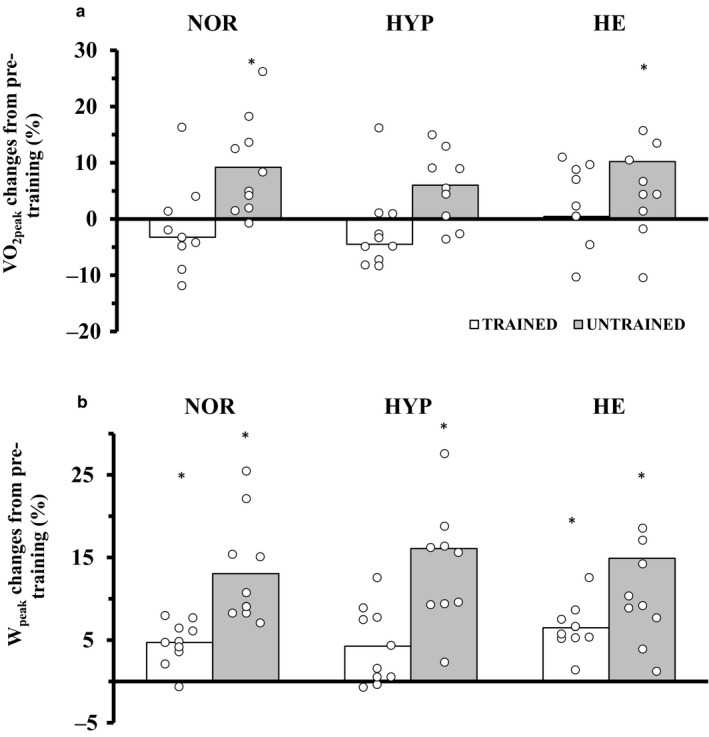
Peak aerobic power (mL·min^−1^·kg^−1^, A — upper panel) and peak power output (W·kg^−1^, B — lower panel) percentage average (bars) and individual (circles) changes from the pretraining trials in all environmental conditions for the trained (white bars, n = 10) and the untrained individuals (grey bars, n = 10). *p < 0.05 versus pretraining within environmental conditions. A three‐way ANOVA was performed

**Figure 2:**
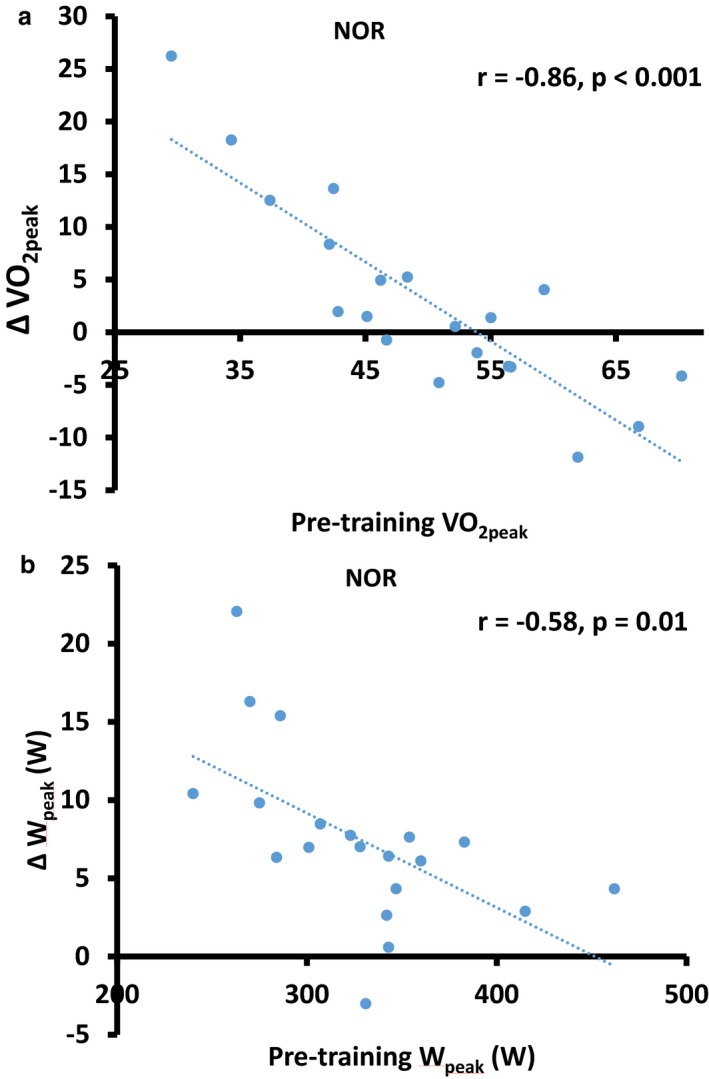
Changes in VO_2peak_ (A) and W_peak_ (B) as a function of respective baseline values in thermoneutral normoxic conditions (NOR, n = 20)

Figure 1a presents percentage changes in relative VO_2peak_ after the 10‐day training period in both groups. A time*group interaction effect was observed for VO_2peak_ (*p* < .001). In the LF group, VO_2peak_ increased in NOR and HE by 9.2 (8.5)% (3.4 [2.7] ml**·**kg^−1^ **·**min^−1^, *p* = .024) and 10.2 (15.4)% (3.2 [4.6] ml**·**kg^−1^ **·**min^−1^, *p* = .037), respectively, but not in HYP (6.0 [5.9]% *p* = .45). VO_2peak_ remained unchanged following the training protocol in the MF group across environmental conditions (*p* > .05). A “very large” correlation (*r* = −.86, *p* < .001) was noted between the initial fitness level and ΔVO_2peak_ in NOR (Figure 2a). Weaker negative correlations were observed between initial fitness level and ΔVO_2peak_ in HYP (*r* = −.53, “large”, *p* = .016) and HE (*r* = −.43, “moderate”, *p* = .067). Similar effects were observed for VO_2peak_ expressed in absolute values (L/min).

Figure 1b presents percentage changes in W_peak_ (normalized to body mass) after the 10‐day training period in both groups. Similar to VO_2peak_, another time*group interaction effect (*p* = .018) was observed for W_peak_ which increased by 13.1 (6.4)% (*p* < .001), 16.1 (9.7)% (*p* < .001), and 14.9 (16.0)% (*p* < .001) in NOR, HYP, and HE, respectively, in the LF group. MF participants did not benefit to the same degree with mean increases reaching 4.7 (2.6)% (*p* < .001), 4.3 (4.6)% (*p* = .019), and 6.5 (2.9)% (*p* < .001) in the respective environments. Again, “large” correlations were noted between baseline W_peak_ and ΔW_peak_, both expressed in W, NOR (*r* = −.58, *p* = .010, Figure 2b), and HYP (*r* = −.52, *p* = .018). ΔW_peak_ in HE was not correlated with baseline W_peak_ (*r* = −.36, *p* = .13).

Several variables that are associated with aerobic exercise performance are depicted in Table 2. A time*group interaction effect (*p* = .046) was only reflected in a higher CO_max_ by 15.4 (12.5)% (+3.2 [2.4] L/min, *p* = .011) in the LF group in HYP. Similar to VO_2peak_, the initial fitness level was “moderately” correlated with ΔCO_max_ in NOR (*r* = −.48, *p* = .038) but not in HE (*r* = −.16, *p* = .52). Surprisingly, no main effect of group was observed even when CO_max_ was normalized to body surface area (*p* = .25). Furthermore, HR_peak_ was lower (*p* < .01) and SV_peak_ was higher (*p* < .001) post‐training across groups/environmental conditions. As expected, VT2 (expressed in ml·kg^−1^·min^−1^) was higher in MF individuals (main effect of group, *p* < .001). Peak ventilation was augmented post‐training only in the LF individuals across environmental conditions (time*group interaction effect, *p* = .002).

### 3.3 Cardiorespiratory responses during submaximal exercise

Table 3 provides cardiorespiratory responses during the 30‐min steady‐state exercise in both MF and LF groups. Post‐training HR was lower across environmental conditions and training groups (*p* < .05). CO was lower (main effect of environmental condition: *p* < .001) in NOR by at least 1.5 l·min^−1^ compared with HYP and HE across training groups. Expectedly, SV was enhanced post‐training in HYP (+17 [10] mL, *p* = .010) in the LF participants, whereas the same tendency was observed in NOR (+14 [9] ml, *p* = .070). A main effect of time was observed on GME (*p* = .002). However, the post hoc tests only revealed an increased GME in HYP for MF individuals (*p* = .027). Minute ventilation was higher in the MF participants compared with their LF counterparts (*p* < .01). Across groups and environmental conditions, participants were rating the exercise workload as lighter post‐training (*p* < .001). S_p_O_2_ was higher in NOR and HE compared with HYP across time and groups (*p* < .001).

**Table 3:** Cardiorespiratory responses and RPE during constant‐work rate cycling pre‐ and post‐training for the normoxic, the hypoxic, and the trial in the heat for the more fit (*n* = 10) and the less fit group (*n* = 10)

More Fit	Pretraining			Post‐training		
	NOR	HYP	HE	NOR	HYP	HE
HR (bpm)	141 (9)	154 (12)	154 (14)	132 (12)^*‡†^	144 (10)*	143 (8)*
CO (L·min^−1^)	15.4 (3.3)	17.8 (2.7)	17.2 (2.4)	15.7 (3.1)	17.5 (3.0)	17.3 (2.6)
SV (ml)	110 (25)	116 (18)	112 (14)	119 (24)	122 (23)	121 (17)
VO_2_ (ml·min^−1^)	2,299 (297)	2,373 (258)	2,279 (222)	2,234 (260)	2,194 (227)*	2,298 (297)
GME (%)	19.1 (1.6)	18.4 (1.2)	19.4 (3.0)	19.5 (1.6)	19.7 (1.4)*	19.1 (1.9)
V_E_ (L·min^−1^)	55.2 (6.7)‡	72.6 (7.5)†	56.9 (7.1)	53.4 (6.6)‡	66.9 (8.6)^*†^	57.0 (8.2)
S_p_O_2_ (%)	99 (95–100)	81.5 (72–88)	99 (97–100)	98.5 (98–100)	82 (77–85)	99 (98–100)
RQ	0.90 (0.02)	0.90 (0.03)	0.88 (0.04)	0.91 (0.02)	0.94 (0.04)	0.90 (0.04)
RPE	13.4 (2.6)	14.9 (1.9)	13.4 (1.8)	10.8 (1.6)^*‡^	13.1 (1.7)	11.4 (2.0)*
Less Fit						
HR (bpm)	136 (10)‡†	158 (9)	157 (11)	123 (13)^*‡†^	144 (11)*	144 (10)*
CO (L·min^−1^)	14.8 (1.9)	16.8 (2.6)	17.3 (3.0)	14.6 (2.8)	17.8 (3.4)‡	16.3 (3.0)
SV (ml)	107 (15)	106 (14)	107 (26)	120 (20)	123 (20)*	113 (28)
VO_2_ (ml·min^−1^)	1875 (244)	1973 (263)	1953 (278)	1863 (269)	1872 (269)	1915 (305)
GME (%)	19.4 (1.9)	18.2 (0.9)	18.0 (1.4)	19.4 (1.1)	19.2 (0.8)	19.0 (0.8)
V_E_ (L·min^−1^)	44.3 (5.7)†#	61.1 (9.4)^#^	48.9 (7.5)	45.5 (6.0)†	55.9 (9.8)^*#^	47.2 (7.1)
S_p_O_2_ (%)	99 (98–100)	84 (76–89)	99 (97–100)	99 (97–100)	84.5 (70–89)	99 (98–100)
RQ	0.87 (0.03)	0.91 (0.04)	0.89 (0.02)	0.89 (0.03)	0.95 (0.05)	0.87 (0.04)
RPE	12.7 (2.4)	14.4 (2.8)	14.3 (2.9)	8.8 (1.6)^*†^	11.5 (2.3)*	10.2 (2.0)*

Note

Values are Mean (*SD*) except for S_p_O_2_ median (range).

* Significant difference to values before HA.

^†^ Significant difference to heat values.

^‡^ Significant difference to hypoxic values.

^§^ Significant difference to More Fit participants, *p* < .05.

### 3.4 Thermoregulation

An overview of the thermoregulatory variables during the 30‐min submaximal exercise is shown in Table 4. A tendency for a time*group interaction effect (*p* = .10) was reflected in a tendency for the resting T_re_ to be lower post‐training in HE only in the MF participants (37.24 [0.19] vs. 37.02 [0.23]℃, *p* = .089). MF individuals demonstrated a tendency for a lower resting T_re_ (main effect of group: *p* = .10) as compared with LF individuals, whereas participants across groups had a lower resting T_re_ post‐training (main effect of time: *p* = .005). In HE, MF individuals increased their T_re_ ~ 0.5℃ more than the LF (*p* < .01). A similar tendency was observed in HYP pretraining (*p* = .060). Whereas ΔT_re_ was consistently higher (*p* < .05) in HE compared with NOR for MF participants, ΔT_re_ was not affected by environmental condition in the LF participants (*p* > .05). T_sk_ was higher (*p* < .001) in HE compared with NOR and HYP across time and participant groups. T_sk_ remained lower post‐ compared with pretraining across environments (~0.2℃ in HE to ~ 0.4℃ in NOR and HYP, *p* < .001). A main effect of environmental condition (*p* < .001) on ΔT_f‐f_ indicated that individuals across groups demonstrated a higher peripheral perfusion when exercising in HE.

**Table 4:** Thermoregulatory responses at rest and during the constant‐work rate cycling pre‐ and post‐training for the more fit (*n* = 10) and less fit (*n* = 10) groups in normoxic, hypoxic, and the heat trials

More Fit	Pretraining			Post‐training		
	NOR	HYP	HE	NOR	HYP	HE
T_re rest_ (℃)	37.29 (0.26)	37.23 (0.18)	37.24 (0.19)	37.12 (0.29)	37.03 (0.19)	37.02 (0.23)
ΔT_re_ (℃)	0.64 (0.12)	0.84 (0.25)	0.98 (0.33)	0.66 (0.15)†	0.69 (0.21)	0.86 (0.24)
H_prod_ (W/kg)	8.4 (0.8)	8.8 (0.7)	8.3 (0.9)	8.2 (0.8)	8.0 (0.5)*	8.4 (0.8)
T_sk_ (℃)	33.8 (0.7)†	33.6 (0.5)†	36.3 (0.3)	33.7 (0.4)†	33.6 (0.5)†	36.1 (0.3)
ΔΤ_f‐f_ (℃)	3.3 (3.5)†	3.6 (4.2)	0.4 (1.2)	4.7 (3.6)†	4.4 (3.6)†	0.2 (1.3)
ṁ_sw,peak_ (g·m^−2^·min^−1^)	1.16 (0.34)	0.90 (0.40)	1.14 (0.47)	0.97 (0.37)†	0.91 (0.33)†	1.49 (0.33)
ṁ_sw_ gain (g·m^−2^·min^−1^·℃^−1^)	1.20 (0.70)	1.06 (0.45)	1.16 (1.10)	1.16 (0.52)	1.02 (0.46)	1.48 (0.62)
H_prod_ (W/m^2^)	326 (36)	340 (30)	321 (35)	316 (31)	312 (24)*	325 (36)
Whole‐body sweat rate (L)	0.60 (0.10)†	0.60 (0.15)†	0.81 (0.14)	0.63 (0.11)†	0.57 (0.15)†	1.01 (0.28)
H_prod_ (W)	640 (87)	666 (70)	629 (78)	620 (77)	611 (64)*	639 (90)
Threshold T_re_ for sweating (℃)	37.52 (0.24)	37.41 (0.16)	37.38 (0.16)	37.28 (0.29)	37.16 (0.21)	37.10 (0.18)*
Less Fit						
T_re rest_ (℃)	37.27 (0.37)	37.33 (0.31)	37.35 (0.21)	37.30 (0.29)	37.21 (0.15)	37.26 (0.24)
ΔT_re_ (℃)	0.37 (0.21)	0.49 (0.20)	0.48 (0.23)§	0.42 (0.20)	0.46 (0.15)	0.37 (0.13)§
H_prod_ (W/kg)	6.1 (0.6)§	6.6 (0.6)§	6.6 (0.8)§	6.1 (0.7)§	6.2 (0.6)§	6.3 (0.5)§
T_sk_ (℃)	33.4 (0.5)†	33.3 (0.8)†	36.2 (0.3)	33.2 (0.6)†	33.5 (0.6)†	35.8 (0.3)
ΔΤ_f‐f_ (℃)	2.1 (3.3)	1.9 (2.5)†	0.2 (1.8)	1.4 (3.4)	3.8 (2.1)†	0.2 (1.5)
ṁ_sw,peak_ (g·m^−2^·min^−1^)	1.08 (0.43)	0.88 (0.19)	1.20 (0.21)	0.70 (0.25)†	1.00 (0.35)	1.28 (0.39)
ṁ_sw_ gain (g·m^−2^·min^−1^·℃^−1^)	2.59 (1.20)	1.73 (0.77)	2.32 (1.22)	1.42 (0.91)	1.59 (1.00)	2.10 (1.46)
H_prod_ (W/m^2^)	252 (22)§	271 (19)§	270 (28)	251 (25)§	254 (22)§	258 (24)§
Whole‐body sweat rate (L)	0.46 (0.12)†	0.46 (0.07)†	0.69 (0.10)	0.56 (0.12)†	0.50 (0.12)†	0.81 (0.13)
H_prod_ (W)	517 (71)	557 (74)	549 (83)	515 (76)	522 (76)	530 (83)
Threshold T_re_ for sweating (℃)	37.41 (0.24)	37.45 (0.31)	37.38 (0.17)	37.46 (0.18)	37.33 (0.13)	37.31 (0.25)

Note

T_re_ resting rectal temperature, ΔT_re_ difference from resting value in rectal temperature, H_prod_ metabolic heat production, T_sk_ skin temperature, ΔΤ_f‐f_ difference between forearm and fingertip skin temperatures, ṁ_sw_ sweating rate. Values are mean (*SD*).

* Significant difference to pretraining values.

^†^ Significant difference to heat values.

^‡^ Significant difference to hypoxic values.

^§^ Significant difference to More Fit participants, *p* < .05.

Main effects of time and group as well as environmental condition*time interaction effects were observed for metabolic heat production (H_prod_), H_prod_ normalized to body weight (W/kg), and H_prod_ normalized to body surface area (W/m^2^) (*p* < .05). Following the post‐training increase in GME in MF people in HYP, H_prod_ was lower (*p* < .01) in that condition post‐training in MF individuals. However, only when expressed in W/kg (*p* < .01) or W/m^2^ (*p* < .05), H_prod_ was consistently lower in LF versus MF individuals across trials. Whole‐body sweat rate was higher (*p* < .01) in HE trials across groups and time. Despite the fact that there was a main effect of time (*p* = .011), the increases of 0.19 (0.25) L (*p* = .11) for the MF and 0.12 (0.12) L (*p* = .19) for the LF individuals in HE post‐training did not reach statistical significance.

**Figure 3:**
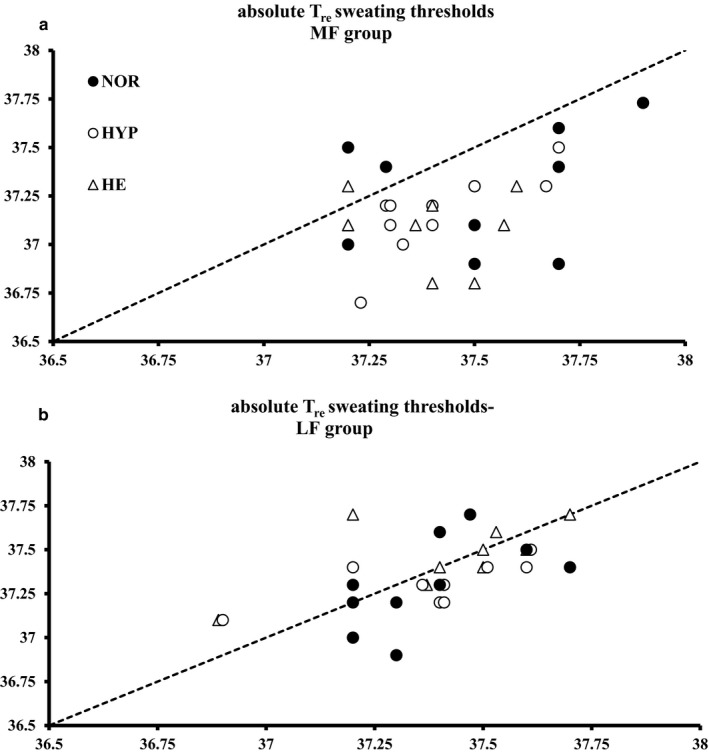
Pre‐ and post‐training individual data for absolute Tre sweating thresholds in normoxia (closed circles), hypoxia (open circles), and heat (triangles) in the MF (a – upper panel, *n* = 10) and the LF (b – lower panel, *n* = 10) individuals. A three‐way ANOVA was performed

Figure 3 presents individual data against the line of no difference (before and after the 10‐day training period) for T_re_ sweating threshold in both groups. A time*group interaction effect (*p* = .003) is reported for the sweating threshold—expressed in absolute values—and is further reflected in post‐training decreases by 0.29 (0.26)℃ (*p* = .028) and 0.26 (0.14)℃ (*p* = .08) in HE and HYP, respectively, but not in NOR (0.24 [0.32]℃, *p* = .12) for the MF individuals. When sweating thresholds were expressed as relative values, i.e., changes from the resting T_re_, participants initiated sweating at a lower ΔT_re_ in HE (*p* < .01) and LF participants sweated earlier than their MF counterparts (main effect of group, *p* = .043). A main effect of group for the gain of the sweating response (*p* = .008) indicated a higher thermosensitivity for the LF individuals.

### 3.5 Hematology

Table 5 presents the hematological marker responses measured over the course of the training protocol. Main effects of time were observed for Hct (*p* < .001), Hb concentration (*p* < .001), and estimated plasma volume (*p* < .001). Specifically, Hct decreased below baseline values already by day 2 in the MF (*p* = .030) and by day 6 in the LF (*p* < .001) participants. With pretraining values as a reference, Hb concentration was lower already from day 6 in both MF (*p* < .01) and LF (*p* < .01) groups. Based on the above, plasma volume expansion reached statistical significance on day 6 in the MF (*p* < .001) and LF individuals (*p* < .001). Red cell volume remained unaffected across groups (main effect of time, *p* = .16). EPO concentration increased by day 6 in the LF people (*p* = .009) but returned to baseline post‐training (*p* = .48). In contrast, the MF group demonstrated a tendency for lower EPO concentration post‐training compared with baseline values (*p* = .074). The training protocol did not affect intracellular HSP70 in the MF (*p* = .43) or LF individuals (*p* = .69). Similarly, HSP90 remained unchanged over the training days in MF (*p* = .36) and LF (*p* = .145) individuals.

**Table 5:** Resting hematological variables pre‐, on days 2 and 6, and post‐training for the more fit (*n* = 10) and the less fit individuals (*n* = 10)

More Fit	Pretraining	Day 2	Day 6	Post‐training
Erythrocytes (10^12^·L^−1^)	5.1 (0.3)	4.9 (0.3)	4.7 (0.4)*	4.8 (0.3)*
Hb (g/L)	155 (6)	150 (6)	146 (8)*	149 (6)*
Hct (%)	44.7 (1.9)	42.9 (2.1)*	41.8 (2.6)*	42.7 (1.6)*
Plasma EPO (mIU/ml)	11.1 (0.02–156.1)	14.9 (0.9–140.8)	21.7 (4.2–127.4)	4.5 (0.02–32.2)
HSP72 (ng/ml)	0.64 (0.20–3.25)	1.19 (0.20–4.54)	1.32 (0.20–7.32)	0.56 (0.28–8.44)
HSP90 (ng/ml)	0.08 (0.01–1.20)	0.23 (0.02–3.59)	0.19 (0.05–0.70)	0.09 (0.02–1.28)
Plasma volume (%)	–	9.4 (5.7)	16.2 (11.2)*	10.7 (7.2)*
Less Fit				
Erythrocytes (10^12^·L^−1^)	5.1 (0.3)	4.9 (0.3)	4.8 (0.4)*	4.8 (0.3)*
Hb (g/L)	154 (11)	150 (9)	146 (10)*	148 (7)*
Hct (%)	44.3 (3.0)	42.8 (2.6)	41.5 (3.1)*	42.1 (2.5)*
Plasma EPO (mIU/ml)	3.4 (0.01–22.9)†	3.1 (0.01–25.2)†	20.1 (5.0–39.3)*	0.3 (0.01–23.6)^*†^
HSP72 (ng/ml)	0.67 (0.20–4.12)	2.16 (0.20–3.36)	1.81 (0.20–5.57)	0.46 (0.20–6.86)
HSP90 (ng/ml)	0.02 (0.01–0.89)	0.32 (0.04–1.60)	0.42 (0.05–2.96)	0.08 (0.01–3.43)
Plasma volume (%)	–	7.3 (10.0)	14.8 (8.7)*	12.2 (10.8)*

Note

Values are Mean (*SD*) except for plasma EPO, HSP72, and HSP90 data which are presented as median (range).

Abbreviations: Hb hemoglobin, Hct hematocrit, EPO erythropoietin, HSP72 heat‐shock protein 72, HSP90 heat‐shock protein 90.

* Significant difference to pretraining.

^†^ Significant difference to More Fit individuals, *p* < .05.

## 4 DISCUSSION

This study investigated the effect of 10‐day moderate‐exercise training in thermoneutral normoxic conditions on aerobic performance indices and thermoregulation in normoxic, hot, and hypoxic environments. The main finding is that the employed 10‐day moderate‐intensity (50% W_peak_) training protocol increases peak aerobic power, but not gross mechanical efficiency in the less fit individuals in thermoneutral normoxic and hot conditions. Training‐induced thermoregulatory gains appear to be substantial in participants of a high fitness level. Despite evidence of a cross‐over effect of training on performance in temperate and hot environments, there was no such evidence of cross‐adaptation in hypoxia.

### 4.1 Aerobic capacity

It is well established that exercise training elicits several cardiovascular adaptations that contribute to improvements in aerobic performance. Whether tangible benefits can be observed after only ten 60‐min training sessions of a relatively low training intensity remains unresolved. A training effect can be defined as any physiological adaptation that enhances aerobic performance. Given that aerobic performance is determined by VO_2peak_, exercise economy, and lactate threshold (Bassett Jr & Howley, 2000), a training effect should be translated to an improvement in any of these indices. It should be emphasized that for the purpose of investigating a cross‐over effect of training on performance in hot and hypoxic environments, we utilized an exercise intensity that is normally used in acclimation studies, and not an intensity that would be used to provide benefits in aerobic capacity. Nevertheless, we observed a net VO_2peak_ increase of 9.2% in NOR and 10.2% in HE in LF individuals after the completion of 10 daily training sessions. Hickson, Hagberg, Ehsani, and Holloszy (1981) trained recreationally active humans and reported a higher VO_2peak_ after only 18 training sessions. In a similar fashion, Montero and Lundby (2017) demonstrated an average VO_2peak_ increase of ~150 ml in untrained participants following 12 training sessions (2 training sessions per week for 6 weeks) at 65% W_peak_, an intensity higher than the one prescribed in this study. In contrast, Daussin et al. (2007) were unable to detect VO_2peak_ increases in sedentary individuals using a traditional continuous‐training protocol with the exercise intensity at 60% W_peak_. The same participants improved their VO_2peak_ when the same amount of work was performed in interval sessions of higher intensity. The results of this study were not surprising as the continuous‐training group trained as little as 20 min per session. Moreover, recent HeA/HyA studies that involved control groups training for 10–28 days in normoxic thermoneutral conditions failed to demonstrate any ergogenic effect (Karlsen et al., 2015; Lorenzo et al., 2010; Siebenmann et al., 2012). In light of the present results, these findings might be attributed to the different fitness level of the participants as in the acclimation studies individuals were on the upper end of the training spectrum. This possibility is further supported by the “very large” correlation between initial fitness level and training‐induced VO_2peak_ gains reported in this study.

From a mechanistic standpoint, VO_2peak_ is governed by arterial oxygen delivery and extraction. In normoxic conditions, arterial oxygen delivery is constrained by CO_max_. The blood volume expansion observed in both groups already by day 6 could theoretically increase the preload of the heart via the Frank‐Starling mechanism and subsequently lead to higher SV_peak_ and CO_max_ values (Bonne, Doucende, et al., 2014a). Provided that MF individuals possess a higher baseline plasma volume, LF individuals would be expected to benefit more from such a hematological adaptation (Coyle, Hopper, & Coggan, 1990). The respective average CO_max_ increases of 2.1 and 1.4 L/min in NOR and HE for the LF group support this argument, especially in HE where a larger amount of blood is redirected to the skin (as indirectly evidenced by high T_sk_ and low ΔT_f‐f_ values). In HYP, arterial oxygen content is the limiting factor for VO_2peak_. Despite a lower Hb concentration post‐training, CO_max_ was enhanced by +3.2 L/min counteracting the fall in arterial oxygen content. Along the same line, the contribution of an enhanced arteriovenous oxygen difference to VO_2peak_ gains has been emphasized over time (Ekblom, Astrand, Saltin, Stenberg, & Wallstrom, 1968; Murias, Kowalchuk, & Paterson, 2010). Our inability to evoke clear hemodynamic adaptations in the admittedly short time frame might be explained by the low training intensity, as studies that succeeded in elevating CO_max_ (Daussin et al., 2007; Ekblom et al., 1968; Helgerud et al., 2007) elicited HR above 90% of maximal values even for short intervals.

Whether HeA protocols could potentiate an increase in mechanical efficiency (Sawka et al., 1983; Sotiridis, Debevec, et al., 2019a) or not (Karlsen et al., 2015) as measured in thermoneutral conditions is still under debate. The increases in mechanical efficiency following HeA protocols have been argued to be a consequence of either participants’ lower fitness level or familiarization with the experimental set‐up (Nybo & Lundby, 2016). Submaximal oxygen uptake was not altered in the LF group rendering a potential influence of a 10‐day training protocol on GME in NOR unlikely. Sawka et al. (1983) also reported an unaltered metabolic rate after relatively MF participants underwent 4 weeks of physical training program at 75% VO_2peak_. Nevertheless, the oxygen uptake was lower in the same participants when a 10‐day HeA proceeded physical training. These data further reinforce the possibility that any HeA‐induced increase in GME might be attributed to an interactive effect of the heat and exercise stimuli (Sotiridis, Debevec, et al., 2019a) and could be explained by a Q_10_ effect; a lower exercise T_re_ resulting in a lower metabolic rate. In this study, exercise T_re_ remained unchanged and consequently, metabolic rate should not have changed either. Paradoxically, an enhanced GME together with a blunted minute ventilation suggests that MF participants limited their work of breathing when exercising in HYP post‐training.

Expectedly, VT2 did not change either as an exercise intensity higher than 70% VO_2peak_ seems necessary for the elevation of the (ventilatory) anaerobic threshold (Poole & Gaesser, 1985). The “large” correlation observed between baseline values and subsequent increases in W_peak_ is another indication of the differential effects of the selected training protocol in participants of a different fitness level. Given that VT2 remained unchanged, an augmented anaerobic capacity could mediate the improvements in W_peak_ in MF individuals. A learning effect seems rather unlikely as by including a preliminary test, practice gains seem to be limited in repeated performance tests independent of the athletic status of the individuals (Hopkins, Schabort, & Hawley, 2001). Taken collectively, the so‐called “training component” claimed to be present in previous acclimation protocols should be evaluated in view of VO_2peak_ rather than VT2 or even GME gains in NOR.

### 4.2 Thermoregulation

Given that almost 80% of the energy expenditure during exercise is converted into heat (Sotiridis, Miliotis, et al., 2019b), it seems reasonable that training even under thermoneutral conditions could impose a substantial thermal burden on humans. HeA protocols utilize a combination of endogenous heat production and environmental heat load to stimulate desired adaptations. In this study, we attempted to delineate the effects of exercise training per se on thermoregulatory adaptations. Our previous studies clearly demonstrate that a hypoxia‐induced higher relative exercise intensity does not alter thermoregulatory responses compared with NOR in unacclimated individuals (Sotiridis, Debevec, et al., 2019a; Sotiridis et al., 2018). MF participants had to overcome a higher ΔT_re_ increase during submaximal exercise. This is not surprising given that ΔT_re_ is determined by H_prod_ divided by body mass (Jay et al., 2011). H_prod_ expressed in W/kg was consistently higher in MF individuals as they were exercising at higher absolute workloads and had a lower body mass. With regards to the sweating response, sweating was initiated at a lower T_re_ post‐training only in the MF participants. Paradoxically, no such change was observed for the LF participants who, in contrast to the MF individuals, had a higher rate of sweat production for any given ΔT_re_. These findings seem in sharp contrast with the long‐held tenet that, following exercise training, humans demonstrate a decreased threshold temperature for the onset of sweating (Roberts et al., 1977) as well as an increased gain of the sweating response (Nadel et al., 1974). Evaporative heat loss has recently been shown to be superior in trained individuals only when a certain threshold of net heat load is exceeded (Lamarche, Notley, Poirier, & Kenny, 2018). Going one step further, it could be speculated that MF participants were training at an intensity high enough to sufficiently stimulate heat loss in each session, whereas LF participants did not reach that threshold. In view of a low ΔT_re_, LF participants might have just not been sufficiently thermally challenged (Tipton, 2019). Indeed, a similar effect has been observed in the adaptive response to HeA, whereby participants who were exercising at a lower H_prod_ displayed a lower reduction in T_re_ during a subsequent heat‐stress test (Corbett, Rendell, Massey, Costello, & Tipton, 2018). Unfortunately, measures of whole‐body sweat loss were not obtained during the training sessions. Even a daily exercise‐induced increase of T_re_ to 38℃ does not seem capable of potentiating the forehead sweating response unless T_sk_ is simultaneously markedly increased, i.e., participants training in hot conditions (Regan et al., 1996). Moreover, the higher gain of the sweating response in the LF group may have come as a result of the analysis used; LF people demonstrated a lower ΔT_re_ which by definition magnifies the gain of the sweating response. As a result, caution should be taken when the classic analysis of the components of the sweating response (threshold, sensitivity, and peak value) is undertaken between groups when differences in the response are relative to ΔT_re_. Jay et al. (2011) have elegantly demonstrated that the gain of the sweating response is not as sensitive an index to distinguish between trained and untrained participants exercising at metabolic heat productions differing as much as ~120 W/m which is double our respective between‐groups difference in NOR (Table 3). Finally, the higher values of whole‐body sweat rate and peak sweat rate in HE across groups reflect the increased T_sk_ in that environmental condition. It has been elegantly shown that for a given T_c_, local thermosensitivity is augmented when T_sk_ increases in 1℃ increments (Nadel, Bullard, & Stolwijk, 1971). Taken together, a rather high net thermal load accomplished by increasing either exercise workload or ambient heat is a prerequisite for thermoregulatory adaptations to occur after such short‐term protocols as reflected in the MF participants.

### 4.3 Hematology

Red cell volume expansion has been claimed to partly account for blood volume expansion following the first ~8–10 training sessions (Sawka, Convertino, Eichner, Schnieder, & Young, 2000). As a result, we are quite confident that the reported plasma volume expansion by day 6, assessed by the changes in Hb concentration and Hct, constitutes a factual phenotypic adaptation and was not overestimated by any erythropoietic effect. Increases in plasma renin activity and antidiuretic hormone may have mediated the plasma volume expansion at such an early stage (Convertino, Brock, Keil, Bernauer, & Greenleaf, 1980). The finding that only LF individuals demonstrated a higher EPO response by day 6 suggests that the EPO response is potentiated in the untrained versus trained state when humans are subjected to a similar exercise stimulus in terms of duration and relative intensity. Alternatively, an increased EPO response may serve to counteract the concurrent decrease in Hct levels which was stimulated by plasma volume expansion (Montero et al., 2017). This possibility, however, is not supported by the fact that the MF people expanded their plasma volume with EPO remaining unaffected and no observed correlation between the changes in Hct and EPO (data not shown). Intracellular measures of heat stress (HSP70 and 90) remained unaffected by the applied training protocol. McClung et al. (2008) have reported increases in the baseline levels of intracellular HSPs following a successful 10‐day HeA protocol comprising daily exercise (treadmill walking) in a hot environment (T_a_ 49°C, 20% RH). Despite a potentially higher T_re_ attained during the training and testing sessions in the MF compared with the LF group, the magnitude of the increases in T_re_ may not have been sufficient to upregulate HSP mRNA transcription (Gibson, Tuttle, Watt, Maxwell, & Taylor, 2016). Adding to the complexity, differences in the HSP mRNA expression between MF and LF individuals have been dissociated from the respective between‐groups differences in intracellular HSPs (Shin et al., 2004). Alternatively, a large proportion of the signaling for gene transcription is unaccounted for by T_c_ alone. Heat‐shock factor—the major regulator of the heat‐shock gene expression—could be activated by increased metabolic stress. Indeed, the cardiovascular strain was similar between groups during exercise sessions. Further research seems warranted to explore whether the short‐term exercise training‐associated thermal stress is capable of inducing cellular adaptations.

### 4.4 Limitations

The main limitation of this study is that groups were not matched for body mass, body fat, and body surface area. Thus, the differential effects of the 10‐day training protocol on thermoregulatory responses, such as the increase in T_re_ during exercise, between groups might be attributed to different baseline physical characteristics. The motivation behind this study was to apply traditional training directives where exercise intensity is prescribed as a certain percentage of W_peak_ or VO_2peak_. Future studies that aim at investigating the effects of exercise training on thermoregulatory function should match metabolic heat produced (and thermal strain experienced) during training sessions across groups of different fitness level. Given that no between‐groups baseline differences were noted in training‐associated hemodynamic characteristics (stroke volume and cardiac output), it might be prudent for future studies to include more trained individuals or elite athletes in the MF group. VT2 was determined noninvasively using respiratory parameters. Notwithstanding its high reliability (Pallares, Moran‐Navarro, Ortega, Fernandez‐Elias, & Mora‐Rodriguez, 2016), the mechanistic relevance of VT2 to the lactate threshold might be disputable.

## 5 CONCLUSIONS

In conclusion, the findings of this study support that the magnitude of the training‐induced aerobic gains is dependent on an individual's initial fitness level. An acclimation‐derived training effect may be substantial in individuals of a lower rather than higher fitness level. This should be taken into account when comparing the efficiency of acclimation protocols. Thermoregulatory benefits were observed in the MF individuals owing to a higher thermal load sustained during the training sessions. In contrast, no cross‐adaptive effect of training was observed on performance in hypoxia.

## CONFLICT OF INTEREST

The authors declare no competing financial interests.

## AUTHOR CONTRIBUTIONS

A.S., T.D., and I.B.M. conceived and designed research; A.S., T.D., U.C., A.C.M., T.M., and J.T.R. performed experiments; A.S. analyzed data; A.S. interpreted results of experiments; A.S. prepared figures; A.S. drafted manuscript; A.S., T.D., U.C., A.C.M., T.M., J.T.R., and I.B.M. edited and revised manuscript; A.S., T.D., U.C., A.C.M., T.M., J.T.R., and I.B.M. approved final version of manuscript.
